# Benign Uroandrological Ecosystem: A Thorough Overview from Single-Cell and Spatial Transcriptomics

**DOI:** 10.34133/research.1438

**Published:** 2026-09-23

**Authors:** Yandong Xie, Junjiang Ye, Jie Wang, Zhouting Tuo, Ruicheng Wu, Dilinaer Wusiman, William C. Cho, Zhaojie Lyu, Qi Zhang, Dechao Feng, Dahong Zhang

**Affiliations:** ^1^ Urology & Nephrology Center, Department of Urology, Zhejiang Provincial People’s Hospital (Affiliated People’s Hospital), Hangzhou Medical College, Hangzhou 310014, Zhejiang, China.; ^2^ Department of Urology, Nanfang Hospital, Southern Medical University, Guangzhou, Guangdong, China.; ^3^ Department of Urology, Tianjin Institute of Urology, The Second Hospital of Tianjin Medical University, Tianjin, China.; ^4^ Division of Surgery & Interventional Science, University College London, London W1W 7TS, UK.; ^5^ Purdue Institute for Cancer Research, Purdue University, West Lafayette, IN 47907, USA.; ^6^ Department of Clinical Oncology, Queen Elizabeth Hospital, Hongkong SAR, China.; ^7^ Department of Urology, Institute of Precision Medicine, Shenzhen Key Laboratory of Male Reproduction and Genetics, Peking University Shenzhen Hospital, Shenzhen 518036, Guangdong, China.; ^8^ Clinical Medicine Research Institute, Zhejiang Provincial People’s Hospital of Hangzhou Medical College, Hangzhou, Zhejiang, China.

## Abstract

Benign urological and andrological diseases (BUADs) represent common yet complex clinical challenges, where the diversity and dynamic nature of their microenvironments substantially influence disease onset and progression. In recent years, the emergence of single-cell RNA sequencing (scRNA-seq) and spatially resolved transcriptomic (SRT) technologies has provided unprecedented insights into the cellular heterogeneity and spatiotemporal architecture of these disease microenvironments. This review outlines current applications of scRNA-seq and SRT in BUADs, with a focus on their utility in mapping cell–cell interactions, uncovering pathological mechanisms, and prioritizing therapeutic targets. Across the upper urinary tract, prostate, bladder, urethra, and male reproductive organs, single-cell analyses reveal that parenchymal cell dysfunction and microenvironmental remodeling are the core pathological processes of disease progression. This review also discusses the limitations of scRNA-seq/SRT and the existing challenges of current research methods and paradigms in BUADs and outlines future research directions and integrative strategies to facilitate clinical translation and precision medicine. Collectively, these up-to-date findings not only deepen the understanding of the microenvironment in BUADs but also provide a scientific foundation for the development of novel therapeutic strategies.

## Introduction

Benign urological and andrological diseases (BUADs) affect organs from the upper to the lower urinary tract and the male reproductive system, including the kidneys, ureters, bladder, prostate, urethra, penis, testes, and epididymis, and can lead to nonneoplastic conditions such as ureteral strictures, urolithiasis, benign prostatic hyperplasia (BPH), urinary tract inflammation, and testicular dysfunction. These conditions markedly impact patient health, quality of life, and societal burden [1,2]. Current therapeutic approaches regarding BUADs are often limited, primarily focusing on symptomatic management, with frequently suboptimal efficacy [3]. The current healthcare phenomenon of quick fixes, shallow thinking, and standardized protocols may further exacerbate the harm of BUADs [4]. While the clinical manifestations of BUADs are diverse, the prevailing research paradigm remains rigid, limited by our incomplete understanding of their complex pathophysiology. Addressing these disease challenges urgently requires moving beyond existing frameworks to seek breakthroughs across multiple dimensions, including technological innovation in sample detection and molecular mechanism exploration. In recent years, researchers have increasingly recognized the pivotal role of the disease microenvironment in disease initiation and progression, with studies on cellular microenvironment yielding substantial insights, particularly in oncology [5–8]. However, compared to urological malignancies, research into the microenvironment of BUADs remains in its infancy, with limited translational innovation, thereby hindering a deeper understanding of their pathophysiology.

**Fig. 1.**
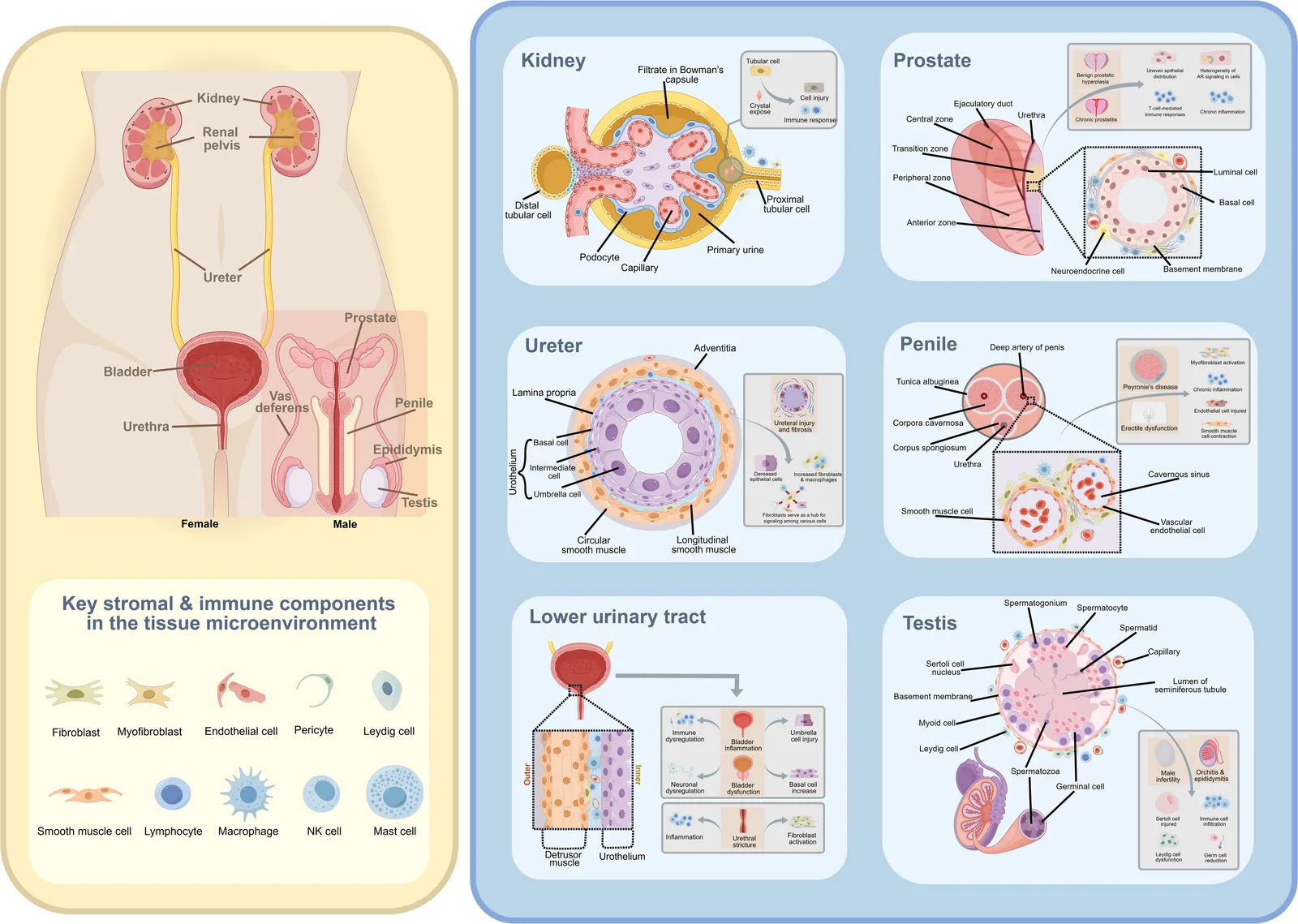
Cellular landscape of the benign genitourinary system. The diagram maps principal cell types across main urinary and male reproductive organs. In the kidney, glomeruli filter blood into primary urine; crystal deposition in tubules triggers tubule cell injury and inflammation, further promoting crystal formation. In the ureter, ureteral stricture tissues show reduced urothelial cells and increased proportions of fibroblasts and inflammatory cells, with fibroblasts’ pro-fibrotic functional activation playing a key role. In inflammatory/functional bladder diseases, umbrella cell damage and increased proportion of basal cells are observed; dysfunction of fibroblasts and immune cells plays important roles in bladder and urethral pathology. In BPH, signaling and distributional abnormalities of epithelial cells may underlie disease progression and treatment resistance; in chronic prostatitis/chronic pelvic pain syndrome (CP/CPPS), evidence from peripheral blood indicates that T-cell-mediated immune responses play a key role. In benign penile diseases, attention should focus on myofibroblasts, smooth muscle cells, endothelial cells, and their interactions with immune cells. In the testicular microenvironment, Sertoli cells are critical for supporting germ cell development and forming the blood–testis barrier, representing a key cell type in benign testicular diseases; interactions among Leydig cells, immune cells, and germ cells also require attention. The figure was created by Figdraw (figdraw.com).

**Fig. 2.**
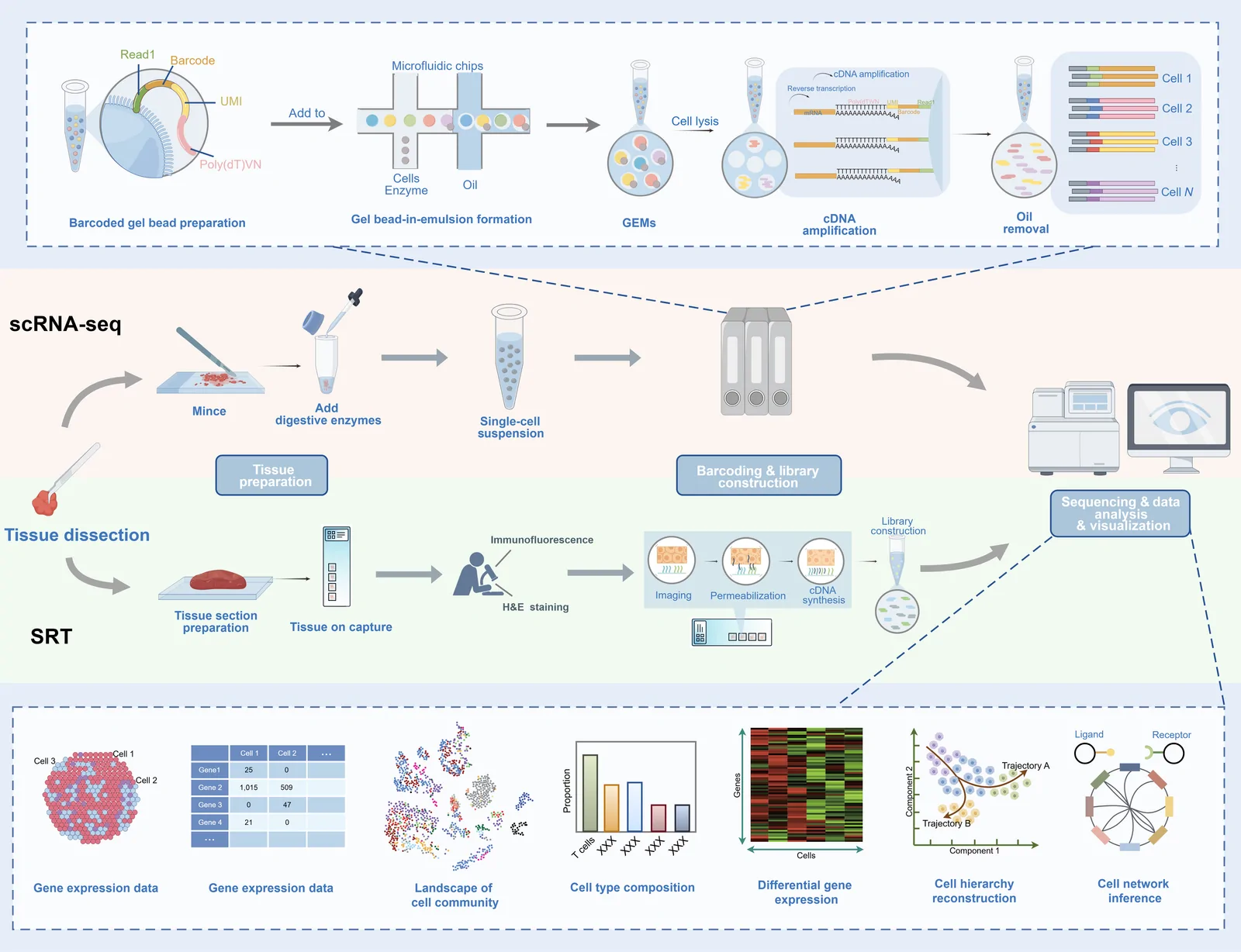
Workflow of single-cell RNA sequencing (scRNA-seq). scRNA-seq is primarily suited for fresh, easily dissociable tissues, whereas spatially resolved transcriptomics (SRT) is suited for structured anatomical units composed of multiple cell types and works with both fresh and (or) formalin-fixed paraffin-embedded tissues. The scRNA-seq workflow using the 10× Genomics’ Chromium platform involves tissue dissociation into single living cells, individual cell barcoding, and RNA capture and amplification, followed by sequencing and bioinformatics analysis to elucidate cell subtype functions. In SRT with the 10× Visium platform, tissue dissociation is not required. Prepared tissue sections are placed on the Visium slide, stained (hematoxylin and eosin [H&E] or immunofluorescence), and imaged and then permeabilized to release messenger RNA (mRNA). The released mRNA binds to spatially barcoded oligonucleotides, followed by complementary DNA (cDNA) synthesis, sequencing library construction, and bioinformatic analysis. The figure was created by Figdraw (figdraw.com).

The urinary system exhibits a diverse yet interconnected cellular composition (Fig. 1). In the kidneys, nephrons filter blood via glomerular capillaries, while tubular cells mediate reabsorption and secretion; urine passes from the renal pelvis via ureters to the bladder, luminal structures composed of urothelium and smooth muscle cells (SMCs) primarily; the urothelium extends to the proximal urethra, transitioning to columnar or squamous epithelium distally; the prostate contains luminal and basal cells lining acini, with a stroma of smooth muscle and fibrous tissue; the penile corpora cavernosa and spongiosum consist of vascular sinuses surrounded by smooth muscle fibers; and testes contain seminiferous tubules with Sertoli and spermatogenic cells, interstitial Leydig cells, and vessels [9,10]. These urinary organs are closely related anatomically and/or histologically, and disruption of their normal cellular structure and functional states underlies BUADs. Cross talk between parenchymal cell dysfunction (altered number/function) in distinct urological niches and chronic inflammation/fibrosis is a core pathological driver of BUAD progression [11–17]. Deciphering these cellular changes at the single-cell level is crucial for understanding BUAD pathophysiology and developing new therapeutic strategies. However, traditional histological and molecular approaches often fall short when it comes to capturing the full complexity of cellular composition, dynamics, and interactions within tissues (Fig. S1). Bulk RNA sequencing, for example, averages signals from mixed populations, while flow cytometry, although capable of single-cell analysis, disrupts spatial integrity and is marker limited. Methods such as immunohistochemistry and in situ hybridization preserve tissue structure but are traditionally restricted to a few targets, offering only a partial view of multicellular interactions [18]. Technologies, including single-cell RNA sequencing (scRNA-seq) and spatially resolved transcriptomics (SRT), help narrow these gaps (Fig. 2) [7]. scRNA-seq platforms such as 10× Genomics’ Chromium enable detailed profiling of cellular heterogeneity and functional states, whereas SRT platforms like Visium retain spatial context to map gene expression within tissue architecture [5,19,20]. From a clinical-translation standpoint, integrating these approaches with bioinformatics can uncover disease-associated cell subsets, potential regulatory pathways, and their spatial dynamics, offering a molecular basis for identifying therapeutic targets and improving patient stratification and prognosis prediction (the core references cited in this review are provided in Table S1).

The application of single-cell sequencing technologies in the study of BUADs is rapidly advancing, having begun to untangle the microenvironmental characteristics of pathological conditions such as BPH and male infertility (MI), demonstrating potential clinical translational value [21,22]. However, compared to the field of oncology, the application of these technologies in BUADs remains substantially lagging. At the level of disease biology, benign lesions lack well-defined recurrently mutated drivers and malignant clonal proliferation, exhibiting heterogeneity in cellular functional states, subtype proportions, and microenvironmental cell–cell interactions, with often obscure pathophysiology [23,24], while tumor mechanism exploration involves numerous hotspot gene pathway loci [25,26]. At the clinical sample level, challenges such as difficulty in obtaining enough typical human lesional tissue and limited tissue quantity in some certain diseases can hinder meeting the requirements for single-cell sequencing [23,27,28]. These factors collectively limit the application of single-cell sequencing in BUADs. Furthermore, at the technical analysis level, limitations of scRNA-seq, including dissociation-induced transcriptome artifacts and dropout events (particularly for lowly expressed genes), introduce large analytical noise and validation hurdles when deciphering the highly complex and subtle biological signals characteristic of the diseases, thereby impacting the accuracy of scRNA-seq [29,30]. Currently, original studies on single-cell sequencing of benign urological organs are scarce (mostly scRNA-seq), with even fewer reviews, and most are limited to a single organ or disease [31–34]. Evidence on DNA- and epigenetic-level regulation of BUAD progression is even more limited. As scRNA-seq and SRT can capture transcriptomic changes in gene expression and cellular function and as BUADs often share similar pathogenic features and anatomic/histologic correlates [17], this review aims to summarize key research findings on scRNA-seq and SRT in nonneoplastic BUADs and to highlight common characteristics across these diseases. It focuses on the critical roles of key cell types and their interactions within the disease microenvironments during pathogenesis and progression, providing novel perspectives and offering research directions for future precision diagnostics and personalized therapeutic strategies.

## Applications of Single-Cell Transcriptomics and Spatial Transcriptomics Technologies in BUADs

In BUADs, the application of scRNA-seq/SRT has untangled a regulatory network spanning urinary organ and disease types. Substantial mechanistic commonalities exist across disease progression in different urinary organs, with parenchymal cell injury and microenvironment remodeling playing profound roles in urinary system disorders (Fig. 1) [17]. For instance, umbrella cells, located in the uppermost layer of the urothelium in the urinary system, form a protective barrier that prevents urine components from penetrating into underlying tissues [10]; their damage or loss can lead to barrier disruption, contributing to conditions such as bladder dysfunction [15,35]. Moreover, the interactions between fibroblasts and immune cells such as macrophages and T cells are important in fibrosis or functional abnormalities of the ureter [36], bladder [15], prostate [37], urethra [38], penis [39], and testis [40]. Furthermore, macrophages display a distinct functional heterogeneity in diseases like renal fibrosis [41] and epididymo-orchitis [40] and engage with fibroblasts, renal tubular cells, and other cell types through ligand–receptor pairs [12,42], forming an intercellular collaborative regulatory network. These findings provide novel insights into the mechanisms of cellular niches in BUADs.

### Upper urinary tract obstructive diseases

Upper urinary tract obstructive diseases primarily include ureteropelvic junction obstruction and ureteral stricture. Developmental defects of the upper urinary tract is an important cause of congenital ureteropelvic junction or ureteral stricture obstruction [43]. Furthermore, acquired ureteral stricture is commonly associated with iatrogenic injuries related to surgery or radiotherapy and can also be induced by benign conditions such as urolithiasis [13]. The pathogenesis of urinary stenotic obstructive diseases is related to the disruption of the urothelial barrier, abnormal activation of pro-fibrotic processes, and excessive deposition of the extracellular matrix (ECM) [13,14]. Currently, surgery remains the primary treatment for upper urinary tract strictures, and there is a lack of effective pharmacological or biological therapeutic strategies to halt or reverse the fibrotic process.

Mechanistic studies on ureteropelvic junction obstruction and ureteral stricture remain limited. In 2022, Fink et al. [44] utilized normal ureteral tissues from 10 patients subjected to radical cystectomy to construct a single-cell and spatial transcriptomic atlas of the human ureter. Trajectory analysis revealed that basal cells with high expression of *Sonic Hedgehog* (*SHH*) serve as the starting point for urothelial differentiation and can differentiate into umbrella cells, via multiple pathways. *COL15A1*^+^ periurothelial fibroblasts were identified as a central hub for cellular signaling interactions, engaging in intensive communication with basal cells through proliferation/differentiation signaling pathways such as WNT and BMP [44]. Moreover, SRT analysis demonstrated that *COL15A1*^+^ fibroblasts are located just below the basal cell layer. Combined with further in vitro organoid validation, these findings suggest that *SHH*^+^ basal cells may represent a potential population of ureteral progenitor/stem cells, while *COL15A1*^+^ fibroblasts were inferred to serve as a critical signaling hub, mediating tissue homeostasis through their interactions with basal cells [44]. However, in ureteral stricture tissue, scRNA-seq and laboratory validation showed that the proportion of urothelial cells (UCs) is highly reduced with functional impairment, and cross talk between injured UCs and fibroblasts is also markedly decreased, which may underlie fibroblast homeostasis dysregulation and aberrant myofibroblast activation [45].

Fibroblasts play a crucial role in scar formation following ureteral injury. Through scRNA-seq of human ureteral scar stricture tissues, Ding et al. [36] found that compared to those in normal ureteral tissues, the proportions of fibroblasts (9.96% vs. 3.38%), macrophages (8.36% vs. 0.54%), and pericytes (10.96% vs. 0.15%) were largely increased in strictured tissues, while the proportion of epithelial cells was markedly decreased (18.5% vs. 94.9%). CellChat analysis revealed fibroblasts as a signaling hub, with enrichment of ligand molecules such as PERIOSTIN and collagen within them. The PERIOSTIN–integrin and collagen–integrin axes constituted communication bridges between fibroblasts and immune cells, including T cells and macrophages. Fibroblasts exhibited high heterogeneity, encompassing inflammatory, ECM-producing, and myofibroblast subtypes. Targeting specific fibroblast subpopulations may help prevent scar formation and restenosis during the repair process [36]. The cellular components and their interrelationships are also complex in the renal fibrosis process associated with ureteral obstruction. To study renal fibrosis from ureteral obstruction, Paul et al. [46] used SRT in murine obstructive kidneys, observing proximal tubular niches enriched for macrophage-derived pro-fibrotic signals. Wang et al. [41] used scRNA-seq in mouse kidney samples following unilateral ureteral obstruction (UUO) and found increased macrophage and neutrophil infiltration. Pseudotime analysis showed a macrophage-to-myofibroblast transition. The expression of the pyroptosis executor *Gsdmd* was elevated in neutrophils and macrophages after UUO, and genes related to neutrophil extracellular traps (NETs) in neutrophils were activated by UUO. Abundant intercellular signaling among macrophages, neutrophils, and myofibroblasts was inferred [41]. Further experiments demonstrated that targeting GSDMD-dependent NET formation in neutrophils inhibited macrophage-to-myofibroblast transformation, thereby reducing renal fibrosis and suggesting a potential treatment strategy for obstructive nephropathy [41].

### Urolithiasis

The formation of urolithiasis begins with crystal growth and aggregation in the urinary tract and their retention at narrow sites. Its pathophysiology involves renal tubular epithelial injury and urinary solute supersaturation leading to nucleation and retention [47]. Crystals can obstruct tubules, causing epithelial damage and interstitial fibrosis while also impairing urinary acidification, further promoting crystallization in a positive feedback loop [47,48]. Currently, the specific cellular and molecular mechanisms behind stone formation and kidney injury remain unclear, and there is a lack of fundamental preventive drugs targeting this process.

Renal tubular cells are the primary parenchymal cell type damaged in kidney injury caused by crystal deposition. Based on scRNA-seq, Duan et al. [49] found that in mouse model of calcium oxalate (CaOx) nephropathy, the proportion of renal proximal tubular cells (PTCs) significantly decreased, and ferroptosis pathways were enriched in PTCs. Subsequent analysis further confirmed through in vivo and in vitro experiments that CaOx crystals induce ferroptosis in PTCs, promoting kidney damage and crystal deposition [49]. Stones can also induce metabolic disorders in renal tubular cells, promoting the occurrence of damage. Yuan et al. [50] performed scRNA-seq on kidney samples from kidney stone patients and identified high expression of *PRMT1* in PTCs. Further experiments showed PRMT1 was up-regulated in kidneys with CaOx-induced injury. Enrichment analysis revealed enrichment of metabolic pathways and the peroxisome proliferator-activated receptor (PPAR) signaling pathway in tubular epithelial cells, while overall tissue key lipid-related pathways were down-regulated in the stone group. Subsequent validation and analysis suggested that PRMT1 inhibits fatty acid metabolism by suppressing PPARγ, thereby promoting CaOx-crystal-induced kidney damage [50]. Renal Randall’s plaque (RP) refers to subepithelial calcium phosphate deposits on the renal papillae that serve as attachment sites for CaOx crystal deposition and stone growth [51]. Recently, a study using scRNA-seq showed widespread down-regulation of *fatty acid-binding protein 3* (*FABP3*) across various renal cell populations within RP [52]. FABP3 deficiency was linked to dysregulation of lipid metabolic pathways, suggesting that lipid metabolic dysregulation may be involved in RP-mediated urolithiasis [52].

Immune response mechanisms associated with crystal retention are key drivers in the progression of urolithiasis. Canela et al. [53] integrated with multi-omics detection technologies, including single-nucleus RNA sequencing (snRNA-seq), SRT, and CODEX multiplexed imaging, to analyze human urolithiasis kidney samples. They found that the proportion of undifferentiated cell clusters was significantly higher in the stone group compared to that in healthy controls. These cells highly expressed injury-associated markers such as *IGFBP7* and *PROM1*. Spatial analysis revealed that undifferentiated cells and inflammatory macrophages were both enriched in RP regions. These regions exhibited high expression of immune activation or matrix remodeling-related genes, including *MMP9*, *CHIT1*, and *SPP1*. Aggregations of T cells and B cells were also observed around the plaques, collectively forming an “immune activation niche”. These findings indicated that mineral deposition may trigger a marked local immune injury response [53]. Jin et al. [12] used scRNA-seq on urolithiasis mouse kidney samples at days 0, 1, 3, 5, and 7 and identified that a subset of injured PTCs highly expressed *HAVCR1*. This subset showed elevated expression of genes related to urinary crystal regulation (like *SPP1* and *FGG*) and pro-inflammatory/pro-fibrotic genes (like *CXCL1* and *PDGFB*). CellPhoneDB2 analysis suggested progressively enhanced SPP1–CD44 interaction between *HAVCR1*^+^ PTCs and monocytes/macrophages. Further subclustering of the monocyte population identified *Fn1*^+^ resident macrophages and *Ly6c*^hi^ infiltrating macrophages, and the CCL2–CCR2 ligand–receptor pair between *Fn1*^+^ and *Ly6c*^hi^ macrophages was strengthened [12]. Subsequent validations and experiments confirmed that resident macrophages recruit infiltrating macrophages via axes including CCL2–CCR2, amplifying the inflammatory response and exacerbating tubular injury. Targeted blockade of CCL2 reduced the accumulation of infiltrating macrophages, alleviated kidney damage, and decreased CaOx crystal deposition [12]. Furthermore, future studies should clarify whether the accumulation of specific inflammatory cell subsets promotes or inhibits stone formation, given that renal medullary macrophages have the potential to remove intratubular particles and suppress local lithogenesis [54].

### Bladder inflammatory disorders

As the storage organ for urine, the bladder has more developed urothelium and muscularis than the renal pelvis/ureter [9,10]. Bladder inflammation is often caused by urinary tract infections, particularly those involving uropathogenic *Escherichia coli* (UPEC) [55]. Recurrent infections, with the emergence of antibiotic drug resistance, may ultimately progress to chronic cystitis [56]. Cystitis glandularis (CG), a common bladder proliferative disorder resulting from chronic inflammatory stimulation, is characterized by the formation of von Brunn’s nests by bladder epithelial cells within the lamina propria [57]. Furthermore, interstitial cystitis/bladder pain syndrome (IC/BPS) represents a heterogeneous group of chronic, sterile inflammatory bladder conditions with an unclear etiology [58]. Its pathogenesis is associated with disruption of the bladder mucosal barrier, dysregulation of the bladder immune microenvironment, and neurogenic inflammation [34,58]. Currently, the clinical management of bladder inflammatory disorders relies mainly on symptomatic treatment and observation, lacking sufficient molecular and cellular evidence for guidance.

IC/BPS is a primary focus of research within bladder inflammatory disorders, with current research centering on how epithelial barrier damage interacts with the bladder immune microenvironment. Peng et al. [59] performed scRNA-seq/SRT on human IC/BPS bladder samples, systematically delineating the immune microenvironment features of IC/BPS. The study revealed a significant increase in the proportions of activated B cells (46.32% vs. 23.99%) and regulatory T cells (Tregs) (18.23% vs. 7.02%), alongside a marked decrease in CD8^+^ effector T cells (17.79% vs. 20.40%) and CD14^+^ macrophages (23.86% vs. 31.60%) in IC/BPS. CellPhoneDB revealed that epithelial cells could activate multiple immune cell subtypes via chemokines CCL15–CCR1/3 and CX3CL1–CX3CR1. Moreover, SRT demonstrated that in IC/BPS, immune cells specifically aggregated around the urothelium and fibroblasts, whereas in control samples, they were predominantly distributed in the urothelium and smooth muscle regions. Pseudotime analysis delineated a differentiation trajectory of macrophages transitioning from a CD14^+^ to an M2-like phenotype. The differentially expressed genes of M2-like macrophages showed substantial associations with autoimmune diseases such as multiple sclerosis and Guillain–Barré syndrome [59]. These results suggest that UCs and fibroblasts play a crucial role in shaping the immune microenvironment in IC/BPS. Furthermore, enrichment analysis identified a virus-related response during the dynamic changes in selected cell types. Moreover, human polyomavirus-2 was detected in the urine of IC patients at a positive rate of 95%, providing clues for the potential role of viral infection in the pathogenesis of IC/BPS [59]. Hunner-type IC/BPS (HIC) is characterized by thinning of the urothelium and a reduced number of umbrella cells, leading to permeation of urine components and stimulation of nerve endings, which triggers pain and inflammatory responses. scRNA-seq of human HIC bladders revealed a significant increase in the proportion of progenitor-like multipotent cells and a decrease in *UPK3A*^+^ umbrella cells (*P* < 0.05) [35]. The core of intercellular communication shifted from SMCs to UCs. *UPK3A*^+^ umbrella cells were enriched in pathways related to immune responses and epithelial repair, particularly the Toll-like receptor (TLR) signaling pathway, and NR2F6 was predicted as a key transcription factor for *UPK3A*^+^ cells [35]. Subsequent in vivo and in vitro experiments confirmed that TLR3 mediates umbrella cell injury and disruption of the bladder barrier function by up-regulating NR2F6, thereby identifying the TLR3–NR2F6 axis as a potential therapeutic target for HIC [35].

In CG, Zhou et al. [60] utilized scRNA-seq to untangle the features of epithelial hyperplasia and immune dysregulation in human CG tissues. On one hand, the proportion of epithelial cells was elevated (71.5% vs. 22.5%), with a particularly profound increase in *PIGR*^+^ epithelial cells. *PIGR*^+^ cells were positioned at the terminal end of the differentiation trajectory, and these cells were enriched in protein secretion and IL-6 inflammatory pathways. During the differentiation process, the expression of superficial epithelial cell markers gradually decreased, while the expression of epithelial–mesenchymal transition (EMT)-related genes increased [60]. On the other hand, the communication between CD8^+^ T cells and natural killer (NK) cells was the most intensive. Cell-trajectory analysis indicated that T cells gradually differentiated from a highly cytotoxic state toward an exhausted state, whereas B cells differentiated from conventional memory B cells toward plasma cells. NK cells were enriched in immune–inflammatory pathways such as IL-2 and IL-6 signaling [60]. Furthermore, InferCNV analysis indicated that CG lacks the typical genomic variations observed in bladder cancer, suggesting a marked distinction between CG and bladder adenocarcinoma [60]. For bladder infections, scRNA-seq can identify dynamic changes in immune cell subpopulations during infection, revealing the activation states and polarization directions, thereby pinpointing key cellular component in the infectious process. Liu et al. [56] utilized scRNA-seq on mouse bladder after UPEC infection and identified a *CD137L*^+^ macrophage subset whose proportion was remarkably elevated in the infected group. This subset, via the CD137L–CD137 signaling axis, activates and enhances the immunosuppressive function of *CD137*^+^ Tregs, thereby negatively regulating the acute inflammatory response, mitigating tissue damage, and limiting bacterial burden. This study revealed that the CD137L^+^ macrophage–CD137^+^ Treg cellular interaction axis is a key mechanism for preventing excessive inflammatory response during bladder infection [56].

### Bladder obstruction/dysfunctional diseases

Bladder outlet obstruction (BOO), often associated with BPH, can coexist with neurogenic bladder (NB) and overactive bladder (OAB), greatly reducing the quality of life [2]. BOO causes high bladder pressure, leading to tissue injury, muscle hypertrophy, and chronic inflammation, which subsequently contribute to bladder fibrosis, nerve impairment, and irreversible dysfunction such as detrusor underactivity (DUA) [15]. NB results from nerve damage, causing detrusor dysfunction and bladder–urethral dyssynergia [61]. OAB involves complicated pathophysiology, reflecting abnormal afferent signaling, leading to bladder dysfunction characterized partly by bladder hypersensitivity, which may be associated with detrusor overactivity [62]. Current treatments for these conditions remain limited.

Chen et al. [15] constructed the first single-cell transcriptomic atlas of obstruction-induced DUA by collecting samples from patients diagnosed via urodynamics with BOO and DUA and controls. The study revealed an increase in the proportions of *RBFOX1*^+^ fibroblasts and *RIPOR2*^+^ macrophages in BOO and DUA groups. Functional analysis indicated that *RBFOX1*^+^ fibroblasts were primarily involved in ECM remodeling, whereas *RIPOR2*^+^ macrophages were enriched in immune regulation and showed a high expression of genes associated with transforming growth factor-β (TGF-β). Moreover, the reduction in umbrella cells and increased proportion in *SHH*^+^ basal cells indicated impairment of the bladder epithelial barrier structure and activation of repair and regeneration programs. Communication among fibroblasts, macrophages, and epithelial cells was the most active, involving key fibrosis- and inflammation-related pathways such as TGFB–TGFBR3 and IL1B–IL1R1 in BOO and DUA groups [15]. Regarding NB-related bladder fibrosis, Wang et al. [42] employed scRNA-seq to dissect the cellular dynamics of rat NB tissues. They found an increase in proliferative fibroblasts and macrophages after bilateral pelvic nerve injury. Further subpopulation analysis revealed a markable expansion of the *Itga8*^+^ fibroblast subset, whose transcriptional profile was enriched in pathways regulating ECM structure and cytoskeleton, suggesting its potential role as a core effector cell driving fibrosis. Concurrently, the *Trem2*^+^ macrophage subset was also largely expanded during the acute phase, with characteristics closely related to ECM and inflammatory regulation. Ligand–receptor interaction analysis demonstrated a strong Fn1–Itga8 axis from *Trem2*^+^ macrophages to *Itga8*^+^ fibroblasts. Pseudotime analysis further indicated that the emergence of *Trem2*^+^ macrophages coincided with the up-regulation of *Fn1* [42]. Subsequent experiments confirmed that the Fn1–Itga8 axis promotes fibroblast activation in NB, with Itga8 playing a central role in coordinating cytoskeletal organization and pro-fibrotic signaling [42]. It should be noted that Trem2^+^ macrophages are usually considered an immunosuppressive myeloid subset, yet their functions vary across diseases [63]. The relationship between their pro-fibrotic effects and their regulation of inflammatory responses requires further investigation.

For OAB, current research efforts are centered on deciphering its main pathogenesis, while studies on local neuro-immune interactions in the bladder remain scarce. Zhang et al. [64] performed snRNA-seq and observed an increased proportion of neurons in the bladders of perimenopausal OAB rat models. Further neuronal subclustering revealed an increase in excitatory and sensory neurons, alongside a decrease in inhibitory neurons in OAB. Moreover, there existed a strong interaction between neurons and macrophages, suggesting that neuro-immune cross talk may be a central node driving the pathological process of OAB. Subsequent in vivo and in vitro experiments further validated the close relationship between neurons and macrophages and demonstrated that macrophages markedly influence neuronal growth and the excitatory (ChAT^+^)/inhibitory (nNOS^+^) neuron ratio [64]. Furthermore, as SMCs constitute the primary functional and structural basis of the bladder detrusor, SRT helps distinguish the spatial distributions among different SMC subtypes and their spatial relationships with other cells like fibroblasts, thereby complementing the mechanistic basis for elucidating detrusor dysfunction [65].

### Benign prostatic hyperplasia

BPH is a benign proliferative disease characterized by overgrowth of both glandular epithelial and stromal cells, primarily in the transition zone (TZ) [9]. This overgrowth can compress the urethra, reducing the pressure gradient between the bladder and the urethra during urination, and is one of the most common causes of BOO [66]. Its pathophysiology involves androgen-dependent cellular proliferation, inflammatory responses, and ECM remodeling, while epithelial–stromal interactions play a critical role [37,66]. However, the disease mechanism remains incompletely understood, and treatment challenges persist, including limited efficacy of anti-androgen therapies and a lack of targeted approaches against stem cells and the inflammatory microenvironment.

BPH nodule formation is a key pathological feature leading to prostate enlargement and lower urinary tract symptoms. Fei et al. [67], by integrating scRNA-seq and SRT data using the robust cell type decomposition method, found that there was a higher proportion of basal epithelial (BE) cells within nodules, while luminal epithelial (LE) cells were located more outside nodules. During nodule formation, epithelial cells transitioned from type 2 LE with strong androgen signaling to type 5 BE cells (BE5) characterized by strong hypoxia signaling and high expression levels of FOS and JUN. These cells ultimately partially transitioned into type 6 BE cells exhibiting high EMT and proliferation signals [67]. The results suggest that BE5 cells and the hypoxia–EMT axis may represent therapeutic targets independent of the androgen pathway [67]. Regarding the differences in disease pathogenesis between the TZ and peripheral zone (PZ), Yan et al. [37] performed scRNA-seq on TZ and PZ tissues from aged human prostate, revealing marked differences in cellular composition and function. The TZ was enriched with *SCGB3A1*^+^ club and *KRT13*^+^ hillock epithelial cells, which highly expressed stem cell markers (like *PSCA*, *LY6D*, and *SOX2*) and NOTCH signaling, while androgen receptor (AR) signaling was lower. Furthermore, the TZ exhibited a higher fibroblast density, increased infiltration of NK cells and Tregs, and greater phenotypic heterogeneity in CD8^+^ T cells and macrophages. In contrast, the PZ was enriched with *TFF3*^+^ LE cells, and the PZ LE cells displayed more active MYC/E2F proliferation pathways, androgen-related signaling, and DNA repair signals, with a relatively stable immune state [37]. Strong ligand–receptor interactions existed between fibroblasts and immune/epithelial cells in the TZ, with the DLL1/JAG1–NOTCH signaling pathway inferred as a potentially key pathway [37].

Cellular senescence plays an important role in BPH progression by driving inflammatory infiltration and fibrotic tissue remodeling in the prostate. Li et al. [21] stratified prostate epithelial cells based on senescence levels using scRNA-seq and gene set variation analysis senescence scoring. The senescence-high epithelial cells significantly up-regulated various senescence-associated genes while also enriching pathways related to the cell cycle and CD4^+^ T-cell recruitment and activation. CellChat analysis revealed increased CXCL signaling interaction between senescent epithelial cells and CD4^+^ T cells [21]. Subsequent experiments demonstrated that the CXCL13–CD4^+^ T-cell axis constitutes a profound immune surveillance pathway, exerting a protective effect and delaying disease progression in BPH [21]. Finasteride is a first-line drug for BPH. To investigate the mechanisms underlying finasteride treatment tolerance, Shen et al. [68] performed scRNA-seq/SRT on BPH tissues from finasteride-treated and untreated groups. The study depicted the changes and spatial distribution of different cell types before and after treatment and found that in the treated group, LE cells were significantly reduced, while BE cells were increased. The AR pathway was markedly down-regulated in epithelial cells overall, without a decrease in *SRD5A2* expression. BE cell proliferative activity was enhanced, whereas AR-mediated transcriptional activity in LE cells gradually decreased, accompanied by an up-regulation of apoptosis-related gene expression. The TGF-β1–EGFR ligand–receptor pair in epithelia was up-regulated posttreatment, and the activity of this pathway positively correlated with cell proliferation markers [68]. This suggests that activation of the TGFB1–EGFR signaling pathway may be one of the potential mechanisms driving prostate proliferation and contributing to clinical tolerance to finasteride [68].

### Chronic prostatitis/chronic pelvic pain syndrome

Chronic prostatitis/chronic pelvic pain syndrome (CP/CPPS) is a noninfectious chronic inflammatory disease of unknown etiology, characterized by persistent inflammatory cell infiltration in the prostate [69]. Its pathophysiology is complex, involving the interplay of multiple factors including immune, inflammatory, neural, endocrine, and metabolic dysregulations [70]. CP/CPPS exhibits marked immune–inflammatory dysregulation, making it an ideal subject for single-cell research.

T-cell-mediated immune responses play a key role in CP/CPPS. Zhang et al. [71] used BD AbSeq to integrate messenger RNA (mRNA) and protein expression profiles from the peripheral blood T cells of CP/CPPS patients. Through clustering analysis based on the multi-omics data, they identified a significantly increased proportion of effector T cells (30.35% vs. 9.38%) in CP/CPPS. Flow cytometry further revealed elevated proportions of central memory T cells and Th1, Th17, and Th22 cells, alongside a significant decrease in Treg cells [71]. Further research discovered that among peripheral blood memory T cells, CD8^+^ T effector memory RA (TEMRA) cells exhibited enrichment in cell signaling and cytotoxicity-related pathways in CP/CPPS [72]. Pseudotime analysis indicated that TEMRA cells were in a transition end state of memory T cells, and there existed enhanced signaling in CD8^+^ TEMRA cells in CP/CPPS [72]. These results suggest that CD8^+^ TEMRA cells may be involved in the pathogenesis of CP/CPPS by driving inflammatory responses. T cells could also promote macrophage activity in CP. Jin et al. [73] found that CCL5 levels were significantly elevated in the peripheral blood of CP patients. Using an autoimmune prostatitis mouse model, they demonstrated that CCL5, by binding to the CCR5 receptor, promotes macrophage polarization toward the M1 phenotype and activates the nuclear factor-κB (NF-κB) and Stat1 signaling pathways, thereby exacerbating inflammation. NF-κB is a critical pathway that regulates myeloid cell differentiation and inflammatory responses [74]. scRNA-seq further revealed predominant expression of *CCL5* in T cells and *CCR5* in monocytes/macrophages/T cells. Moreover, the expression level of *CCR5* was positively correlated with M1 macrophage polarization scores [73]. This indicated that T cells may promote macrophage polarization toward the pro-inflammatory M1 phenotype via the CCL5/CCR5 axis, thereby aggravating CP.

However, it should be noted that the 3 single-cell studies mentioned above used peripheral blood samples, and there is currently a lack of single-cell research on prostate tissue in CP/CPPS.

### Urethra stricture

Although the urethra and bladder are morphologically connected as a continuous urinary tract, spatially distinct genes were identified in the epithelial and mesenchymal compartments [75]. The urethral epithelium serves as a dynamic immunological barrier and is also the primary portal for uropathogens in ascending urinary tract infections [76]. The fibrotic process following urethral injury or inflammation can lead to urethral strictures, characterized by immune cell infiltration and aberrant activation of specific stromal cells [17,77,78]. Buccal mucosa graft (BMG) urethroplasty is one of the mainstay treatments for urethral stricture [79,80]. However, knowledge gaps regarding urethral epithelial cells and resident immune cells restricts the understanding of urethritis pathophysiology and impedes targeted therapy development.

The urethra maintains a tissue-specific immune microenvironment whose cellular and genetic profile enables its role as a first line of defense against ascending infections. Jasmine et al. [76] mapped the immune cell landscape of the mouse urethra using scRNA-seq/SRT. The study identified 2 distinct macrophage subtypes within urethra. Macrophages enriched in the urethral epithelium highly express *Cx3cr1* and MHCII genes, with functional enrichment in pathways related to antigen presentation and inflammatory responses. Meanwhile, macrophages in the urethral stromal layer highly express genes associated with scavenging receptors including *Mrc1* and *Mgl2*, involved in endocytic function, angiogenesis, and tissue homeostasis maintenance [76]. Furthermore, the urethral epithelium expresses monocyte chemoattractants such as *Cx3cl1* and *Cxcl17*, whose expression levels exhibit a gradient increase from the proximal (near the bladder) to the distal urethra. Correspondingly, the distribution density of epithelium-associated macrophages in the urethra shows a similar proximal-to-distal increasing trend [76]. Neuroendocrine cells within the urethra can also exert active defense functions by promoting smooth muscle contraction via serotonin-mediated mechanisms to resist bacterial ascent [81]. Furthermore, Henry et al. [82], utilizing scRNA-seq analysis of human prostatic urethral tissue, discovered enrichment of *SCGB1A1*^+^ epithelial cells in this region. The transcriptional profile of these cells resembles that of club cells in the lung, which possess antimicrobial/immunomodulatory functions, suggesting a potential regulatory role in urethral inflammation [82].

In the context of urethral stricture, elucidating abnormal fibroblast activation and excessive ECM deposition is the current focus. Li et al. [83] performed scRNA-seq on post-traumatic human urethral stricture tissues and adjacent normal urethral tissues, identifying fibroblasts and T cells as the predominant cell proportion in stricture tissues, with a reduced epithelial cell count. Enrichment analysis revealed abnormal activation of fibrosis-related pathways in fibroblasts, and cell-trajectory and SCENIC analyses untangled diversity and heterogeneity in their differentiation states and functions. CellPhoneDB analysis further indicated active interactions between fibroblasts and vascular endothelial cells (ECs), with integrin family molecules (like α10β1 and αvβ3) playing the bridge role in their communications [83]. This suggests that the integrin family may have potential function in regulating urethral fibrosis. For nontraumatic urethral stricture, lichen sclerosus (LS) is an important etiology. Through scRNA-seq, Zhang et al. [38] found that, compared to non-LS urethral stricture tissues, LS-associated urethral stricture disease (LS USD) tissues exhibited significantly increased proportions of myeloid cells, SMCs, and epithelial cells and harbored a specific *SAA1*^+^ fibroblast subpopulation. *SAA1*^+^ fibroblasts were enriched for immune-response-related pathways, and pseudotime analysis suggested that this subpopulation resides at the end stage of fibroblast differentiation. CellPhoneDB analysis further revealed strong interactions of *SAA1*^+^ fibroblasts in LS USD, involving unique interaction with *IL-1B*^+^ macrophages via the SAA1–FPR2 axis. These findings suggested that targeting *SAA1*^+^ fibroblasts may represent a potential novel therapeutic strategy for LS USD [38]. For potential treatment failure after urethroplasty, therapies like low-dose ionizing radiation (LDIR) and low-intensity shock wave therapy (LiSWT) may modulate tissue remodeling. Using SRT in rat models of urethral stricture following BMG urethroplasty, researchers profiled the transcriptional features of distinct tissue regions including the urethra, stroma, and corpora cavernosa, under different treatment conditions and clinical outcomes [84]. SRT revealed that graft dehiscence is associated with transcriptional signatures of inflammation, oxidative stress, and fibrosis, while LDIR up-regulated angiogenic pathways, and LiSWT primarily promoted epithelial remodeling and differentiation, which may improve BMG integration. Key findings were validated by histological analyses in rats. Interestingly, from a spatial transcriptomic perspective, both LDIR and LiSWT showed potential benefits for erectile function [84].

### Peyronie’s disease

The corpora cavernosa and corpus spongiosum of the penis, surrounded by the tunica albuginea, are the key structures for penile erection. The tunica albuginea is mainly composed of dense fibrous tissue. Peyronie’s disease (PD) is a condition characterized by localized fibrosis of the tunica albuginea, manifesting as penile curvature, shortening, and erectile dysfunction (ED) [9,39]. Its pathogenesis is associated with repetitive trauma and chronic inflammation, during which myofibroblasts play a central role in plaque formation, possessing both collagen-producing and contractile functions, leading to tissue fibrosis and contracture [39]. Current research focuses on the transformation mechanisms of myofibroblasts and the biological basis of fibrosis, yet evidence from single-cell sequencing is lacking.

Through scRNA-seq of tunica albuginea plaque tissue from patients with chronic PD, it was found that myofibroblasts constitute the largest proportion of cells in PD (27.8% vs. 8.7%) [85]. The proportions of SMCs, lymphocytes, and myeloid cells were also increased, while the proportion of classical *ACTA2*^−^ fibroblasts was decreased [85]. Concurrently, immune–inflammatory responses were increased in PD. These results suggest the presence of persistent chronic inflammation in PD, and targeting inflammation may be a potential therapeutic strategy for PD [85]. The activation of immune cells and the release of cytokines such as TGF-β1 could promote myofibroblast activation and collagen deposition [39,86]. Since myofibroblasts are ubiquitous in normal wound healing and difficult to target, future research should further utilize single-cell sequencing to explore the activation mechanisms of myofibroblasts in PD, as well as the specific mechanisms of upstream interactions, such as immune cell activation and other fibrosis-related pathways. SRT analysis of penile tissue from PD rats revealed expression of the fibrosis-related gene *Lum* enriched in both fibroblast and EC cluster regions [87]. Lum may promote penile fibrosis via TGF-β pathway activation and thus could serve as a potential interventional therapeutic biomarker for PD [87].

### Erectile dysfunction

The corpus cavernosum (CC) is a vascular sinusoid that engorges during erection. Nitric oxide (NO) from cavernous nerve terminals initiates this process, while endothelial NO sustains it [88]. Within SMCs, NO stimulates cyclic guanosine monophosphate (cGMP) production, lowering intracellular calcium to induce relaxation, increase blood inflow, and reduce venous outflow [89]. ED can result from any disruption in this pathway, with varying mechanisms in conditions such as diabetes, atherosclerosis, and aging. The functional interactions among ECs, SMCs, fibroblasts, and the neurovascular unit in ED are not yet fully understood.

In 2022, the single-cell atlas of the human CC was published [16]. Using CellChat analysis, Zhao et al. [16] found that fibroblasts are the main signal-sending cells in the cavernous microenvironment, while macrophages, Schwann cells, and T cells exhibit typical signal-incoming patterns. ED patients showed a marked increase in the *APOC1*^+^/*PTCHD1*^+^ fibroblast subpopulation, with enriched WNT signaling pathways. In functional experiments, activating WNT signaling induced the up-regulation of muscle-contraction-related and collagen-related genes in fibroblasts and accelerated tissue fibrosis, whereas inhibition of WNT signaling delayed fibrosis [16]. Furthermore, the study revealed that key smooth muscle contraction genes, *MYLPF* and *MYLK*, were significantly up-regulated in ED SMCs, while the Hippo/Yes-associated protein (YAP) signaling pathway, which regulates smooth muscle homeostasis, was profoundly dysregulated. In ED ECs, mitochondrial dysfunction, apoptosis, and TGF-β signaling were up-regulated, suggesting dysfunction in these cells. Intervention targeting the Hippo pathway could alleviate TGF-β-induced endothelial injury and tissue fibrosis [16]. Integrating SRT facilitates the analysis of ED cellular heterogeneity. Yin et al. [28] used scRNA-seq/SRT in human and rat CC and observed a higher proportion of ECs in the cavernous artery region, while areas around the tunica albuginea contained fewer fibroblasts but were rich in SMCs. Spatially, SMCs were located farther from fibroblasts. Arterial ECs, vascular SMCs, Schwann cells, and T cells were found positively correlated spatial patterns with the *COMP*^+^ fibroblast niche, indicating a potential association between *COMP*^+^ fibroblasts and nerves/blood vessels. The *COMP*^+^ fibroblast niche was enriched in terms like “integrin-mediated signaling” and “response to mechanical stimulus”. Using atomic force microscopy and ultrasound elastography, the study showed that *COMP*^+^ fibroblasts were concentrated in high-stiffness regions like the tunica albuginea and septum pectiniforme, whereas *APO*^+^ fibroblasts were enriched in softer areas like the cavernous artery region [28]. These results combined with further experimental validation demonstrate a link between fibroblast subpopulations and spatial variations in ECM mechanical cues.

SMCs are key cells influencing ED and are also affected by mechanical stress signals. A study that performed snRNA-seq on rat penile tissues in the flaccid and erect states found that during erection, the key mechanosensitive pathway YAP/transcriptional co-activator with PDZ-binding motif (TAZ) was up-regulated in SMCs [90]. Further experiments confirmed that mechanical stretching of the penis directly promotes the expression of the vasodilatory peptide adrenomedullin by activating YAP/TAZ, thereby maintaining smooth muscle relaxation. This mechanism is epistatic to the classical NO–cGMP pathway, providing a potential new therapeutic target for ED unresponsive to PDE5 inhibitor treatment [90]. Pericytes are cells surrounding capillary walls, playing a crucial role in maintaining the stability of vascular/neural function. Based on scRNA-seq of normal and diabetic ED cavernosal tissues, Bae et al. [91] identified *LBH* as a specific biomarker for cavernosal pericytes, with its expression significantly down-regulated in ED CC. Cell–cell interaction analysis revealed that interactions involving angiogenesis, adhesion, and migration between pericytes and neighboring cells, such as vascular ECs, were weakened in diabetic conditions. Overexpression of *LBH* demonstrated effective restoration of impaired erectile function and a significant increase in the pericyte, EC, and neuronal cell contents within the cavernosum [91]. Furthermore, single-cell sequencing helps identify critical intervention windows for ED. A study comparing penile tissues from elderly males with mild/severe ED to young normal controls found that most cellular and transcriptomic alterations—including a reduced proportion of ECs, an imbalance in the contractile–relaxation function of SMC, activation of inflammatory/apoptotic pathways in ECs, and a pro-inflammatory shift in the immune microenvironment—had already occurred during the mild ED stage [92]. This suggests that earlier intervention may be critical for ED.

### Orchitis and epididymitis

The spermatogenic microenvironment is a sophisticated network composed of Sertoli cells, Leydig cells, germ cells, immune cells, and others. Homeostatic interactions among these cells collectively maintain spermatogenesis and maturation [93]. Sertoli cells form the main basis of the blood–testis barrier via tight junctions, protecting germ cells from immune attack and providing developmental support, while Leydig cells are responsible for androgen synthesis, which is essential for spermatogenesis [93,94]. Orchitis and epididymitis are important male reproductive diseases. Inflammatory cell infiltration, hypoxia, and oxidative stress can disrupt the spermatogenic microenvironment, impairing testicular and epididymal function.

Bacterial infection can cause alterations in the spermatogenic microenvironment. Zhou et al. [95] employed invasion–adhesion-directed expression sequencing (INVADE-seq) technology to simultaneously capture host and bacterial transcripts within single cells from mouse testes. The study found that normal testicular tissue harbors a widespread but low-abundance bacterial microbiome. The bacterial abundance was higher in cells located outside the blood–testis barrier compared to that in most germ cells within the barrier. In bacteria-positive Leydig cells, steroid hormone synthesis and secretion pathways were up-regulated. Bacteria-positive macrophages exhibited up-regulation of endocytosis/autophagy-related pathways and displayed an M2-like polarization state. Moreover, cell–cell communication activity was enhanced in bacteria-positive cells [95]. Regarding the inflammation, orchitis can badly change spermatogenic cellular function. The scRNA-seq of testicular tissue from mice with chronic autoimmune orchitis revealed increased expression of senescence markers (like *Cdkn1b*) in Leydig and dendritic cells, alongside up-regulated apoptosis-related genes (like *Trp53*) in spermatogonia [96]. Enrichment analysis showed up-regulation of senescence, inflammation, and fibrosis signatures, but down-regulation of steroid synthesis signatures in Leydig cells, with senescence features negatively correlated with the androgen-related pathway [96]. Most pro-inflammatory signals positively correlated with senescence and apoptosis but negatively correlated with androgen and glutathione metabolism, suggesting that glutathione metabolism may be a potential target in testicular inflammatory injury [96]. Additionally, inflammation remarkably altered the outgoing communication patterns by Sertoli cells [96]. Chronic infection and inflammation in the testes/epididymis can lead to fibrosis, affecting spermatogenesis and sperm transport. Wang et al. [40] analyzed immune cells in the testes/epididymis postinfection using scRNA-seq and identified a marked increase in an *S100a4*^+^ macrophage subpopulation. This subpopulation was enriched for fibrosis-related signaling pathways like TGF-β signaling, and S100a4 showed high colocalization with fibrotic areas. Subsequent experiments demonstrated that S100a4 could promote the transformation of macrophages into a myofibroblast-like phenotype. Targeting S100a4 markedly suppressed postinfection immune infiltration, tissue damage, and fibrosis [40].

### Male infertility

The dysregulation of interactions between somatic cells and germ cells during spermatogenesis, dysfunction of the blood–testis barrier, and abnormal immune/inflammatory responses constitute key pathophysiological foundations for germ cell damage, ultimately leading to MI [11].

Aging is a major contributing factor to MI. scRNA-seq analysis revealed that the number of undifferentiated spermatogonia largely decreases in humans in their 50s, and haploid germ cells exhibit substantial alterations in the expression of genes related to flagellar function and DNA repair during aging, indicating cumulative functional damage [97]. Concurrently, various somatic cells (including Sertoli cells, Leydig cells, and macrophages) exhibit marked functional changes, and the proportion of Sertoli cells decreases in the 50s [97]. Aging testes feature elevated inflammation. scRNA-seq/SRT by Huang et al. [98] showed that the Leydig niche has the highest senescence-associated secretory phenotype (SASP) gene set score with a reduction in proportion, while macrophages and Leydig cells are spatially close, with strong interdependencies especially in SASP-high regions. Aged macrophages up-regulate *CCL8*, and functional validation showed that intratesticular injection of CCL8 into young mice induces testicular inflammation, germ cell apoptosis, fibrosis, and steroidogenic dysfunction, mimicking ageing phenotypes [98]. In a single-nucleus transcriptomic atlas of aging testicular in cynomolgus monkeys, it was found that pathways related to cell junction assembly, hormonal response, and WNT signaling were down-regulated in Sertoli cells, while the TGF-β signaling pathway was up-regulated [99]. Moreover, the NRG1–ERBB3 and CADM1–NECTIN3 signaling pathways between Sertoli cells and germ cells were down-regulated, which meant compromised Sertoli cell reservoir and spermatogenesis. These findings combined with experimental validations suggest that aging-related Sertoli cell function impairment may affect blood–testis barrier stability and promote the formation of a microenvironment unfavorable for spermatogenesis [99]. In addition, in type 2 diabetes, scRNA-seq similarly identified a reduction in the expression of genes associated with the blood–testis barrier structure in Sertoli cells, and further experiments confirmed that a high-glucose environment severely compromises the stability of the Sertoli cell blood–testis barrier, thereby disrupting spermatogenesis [22].

scRNA-seq studies on nonobstructive azoospermia (NOA) are relatively abundant. At the germ cell level, gene expression abnormalities related to proliferation and apoptosis have been identified, while at the somatic cell level, Sertoli and Leydig cells also display functional dysregulation [32]. These studies emphasize different cell types. For example, Zhao et al. [100] systematically compared the testicular microenvironments of healthy subjects (from infants to adults) and NOA patients. They found that Sertoli cells were the somatic cell type with the most significant dissimilarity in NOA. Cell-trajectory analysis untangled that normal Sertoli cells undergo 3 consecutive developmental stages: immature, transitional, and mature [100]. In patients with Klinefelter syndrome and AZFa deletion, Sertoli cells could progress to the mature stage, but all exhibited abnormal immune responses (with high expression gene of *macrophage migration inhibitory factor* [*MIF*]). Furthermore, in patients with idiopathic NOA, Sertoli cells were more proliferative but arrested in the immature state, with aberrant activation of WNT/β-catenin signaling [100]. Further validation confirmed that inhibiting the WNT pathway promoted the maturation of idiopathic NOA Sertoli cells and restored their ability to support germ cell survival [100]. Cryptorchidism is an important cause of MI. Through scRNA-seq analysis of testes from adult cryptorchidism patients, Wang et al. [101] found that Leydig/interstitial cells constituted the highest proportion in cryptorchid testes, while germ cells at all developmental stages were generally reduced. Further analysis revealed widespread activation of apoptosis in germ cells, with up-regulated expression of the cell-cycle-arrest-related genes *CDKN1A*, *TP53*, and *RB1* in spermatogonia, and spermatogonial stem cells and differentiating spermatogonia were predominantly arrested in the G1 phase [101]. Concurrently, the proportion of immune cells, particularly mast cells and lymphocytes, was elevated. Mast cells exhibited an activated and degranulated state, and the CTSG–PARD3 ligand–receptor pair was markedly enriched in interactions from mast cells to other germ and somatic cells [101]. This study explains cryptorchidism-related infertility at a cellular level, suggesting that mast cells and their cytokines may be potential therapeutic targets.

## Current Research Summary

Tight and complex interconnections exist among different diseases and cell types in BUADs, while the lack of high-quality mechanistic research in BUADs hinders deeper insight into these diseases. Systematically extracting common cellular mechanisms can deepen our understanding of BUAD pathogenesis and support cross-disease translational strategies. As detailed in the preceding sections, imbalances in the numbers and functional impairments of parenchymal cells—including UCs, PTCs, BE/LE cells, SMCs, Sertoli cells, and germ cells—constitute the pathological foundation for various BUADs, including ureteral stricture, urolithiasis, bladder inflammation/dysfunction, BPH, urethral stricture, and penile/testicular dysfunction [15,16,35,36,45,49,67,83,85,97]. Stromal and immune cells are also deeply involved in the progression of BUADs. In urinary tract stenotic diseases (like ureteral stricture and urethral stricture), the proportion of fibroblasts is markedly elevated, and they actively communicate with immune cells, ECs, and other cell types via pathways mediated by molecules like integrins [36,83]. Single-cell sequencing has identified various fibroblast functional markers in BUADs (including *COL15A1*^+^ [44], *RBFOX1*^+^ [15], *Itga8*^+^ [42], *SAA1*^+^ [38], *COMP*^+^ [28], *APO*^+^ [28], and *APOC1*^+^/*PTCHD1*^+^ [16]) that play potential important roles in BUAD progression, but the specific functions of these subtypes in pan-BUADs require further investigation. The temporary acquisition of a fibroblast-to-myofibroblast phenotype is beneficial for acute wound healing, whereas the persistent maintenance of myofibroblasts drives the development of organ fibrosis [102]. In PD, myofibroblasts constitute the largest proportion of cells and play a critical role [39,85], while macrophages have been shown to transition toward a myofibroblast-like phenotype in obstructive kidney fibrosis [41] and UPEC-infected testes/epididymis [40], promoting tissue fibrosis. Pathways including TGF-β1, S100a4, and WNT/β-catenin signaling participate in the transdifferentiation of the myofibroblast phenotype and play important roles in fibrosis associated with BUADs [16,40,86,103,104], representing important potential pan-organ anti-fibrotic targets. Among macrophage subtypes, *Trem2*^+^, *S100a4*^+^, and *RIPOR2*^+^ subsets show pro-fibrotic capacity in single-cell sequencing analysis, but only S100a4^+^ macrophages have been functionally validated by target knockdown; the therapeutic potential of Trem2^+^/RIPOR2^+^ macrophages as cross-organ targets in BUADs requires further investigation [15,40,42]. Furthermore, in conditions such as urolithiasis, IC/BPS, CG, bladder dysfunction, aged prostate, CP/CPPS, PD, orchitis/epididymitis, and cryptorchidism, various patterns of abnormal activation are observed in different immune cell subsets, including macrophages, T/B lymphocytes, and mast cells [12,15,37,40,42,59,60,71,85,95,101]. In addition, the CXCL13–CD4^+^ T-cell axis plays an active role in eliminating senescent prostate epithelium to delay the progression of BPH [21]. Moreover, cellular senescence exhibits cross talk with apoptosis, immune–inflammatory responses, and cellular functional pathways in BPH, orchitis, and MI, suggesting that it may play an important role in BUAD progression [21,96–99].

However, cross-species and cross-platform heterogeneity may affect our transcriptome-based conclusions. For instance, the SRT platform Visium has been used in studies of the ureter [44], obstructive nephropathy [46], urolithiasis [53], IC [59], BPH [67,68], urethral treatment [84], and testis aging [98]. Visium HD was recently used in bladder research [65], Xenium in urethra [76], and BMKMANU S1000 in penis [28]; in one study on PD, the SRT platform information was not found [87]. Furthermore, different platforms vary in their resolution for tissue transcriptomics [105–107]. Additionally, differences in the species of sequenced tissues may potentially affect the results. In this review of scRNA-seq and SRT studies on BUADs, tissue samples from various species—including human, rat, mouse, and cynomolgus monkeys—are being used. In one study, scRNA-seq data show that the transcriptional similarity of the same cell types between human and rat CC is >70% and the transcriptional profiles were positively correlated, suggesting that using rat models to simulate the transcriptional regulation of the human CC microenvironment is acceptable in most cases [28]. However, the top differentially expressed genes are not shared across species for most cell types, except SMCs, so caution is needed when focusing on limited genes or pathways [28]. To boost reliability and clinical translation, a study combined evidence from rat and mouse models with clinical patient data to validate the crucial role and therapeutic potential of a signaling pathway in ED [90]. Meanwhile, in symptom-based conditions, like OAB, there is a current lack of relevant disease-specific animal models; thus, animal models of detrusor overactivity are mostly used as a surrogate, which may increase the difficulty of dissecting this type of disease [62]. Furthermore, for diseases where organ tissue samples may be difficult to obtain, such as CP/CPPS, using blood as a substitute may also restrict the elucidation of disease mechanisms [71]. Currently, bioinformatics results from single-cell and spatial transcriptomics are primarily preliminary clues and/or computational predictions, with limited evidentiary value and not definitive as conclusions. Nevertheless, the potential targets from global screening provide valuable guidance for the design and implementation of subsequent wet-lab experiments, including histological and functional assays.

## Challenges and Future Prospects

The rapid development of single-cell and SRT technologies provides powerful tools for delineating the microenvironment of BUADs. Nevertheless, several technical constraints hinder their broader application. The complexity of tissue dissociation for scRNA-seq compromises cell viability and function and could induce substantial transcriptomic changes in cell subpopulations [23,30,108]. Library preparation introduces further bias, as technical limitations such as low mRNA capture efficiency result in quantitative distortion of gene expression and dropout events [29]. Quality control steps that filter low-quality cell data based on unique molecular identifier counts, gene numbers, or mitochondrial ratios may exclude specific cell types (such as those with a low metabolic activity, a small size, or active oxidative phosphorylation), which could lead to biased representation [29]. Regarding the extent of tissue dissociation artifacts, it is affected by both the sample type and the chosen dissociation/sequencing strategy. scRNA-seq is well suited for fresh tissues and samples that dissociate readily, such as blood. Furthermore, snRNA-seq prevents stress-related gene expression caused by harsh tissue dissociation by extracting single nuclei through direct lysis of intact tissues. This makes it compatible with a broader range of samples, including frozen tissues, difficult-to-dissociate specimens, and those containing fragile cell types prone to rupture (like nervous system components) [109,110]. However, snRNA-seq cannot capture cytosolic RNA and has relatively lower sensitivity, which may impede the detection of low-abundance transcripts [111,112]. Other strategies, such as SRT, cold digestion, and in situ cell fixation prior to dissociation, can also mitigate tissue-dissociation-induced transcriptome artifacts [111]. SRT does not require single-cell suspension and is suited to structured anatomical units composed of multiple cell types. Formalin-fixed paraffin-embedded tissues are important sample types for SRT [110,113]. Nevertheless, current SRT platforms (like 10× Visium) have a limited detection resolution due to constraints in capture spot size and spacing, mRNA diffusion, limited capture efficiency, and gene-capturing bias [23,105,114]. In the future, with the further development and application of emerging technologies such as Visium HD [115], Stereo-seq V2 [116], CytoSPACE [117], and SpalM [118], transcriptomic tools that can simultaneously provide comprehensive single-cell transcriptomic and spatial information will play a pivotal role in future cellular transcriptomic research. As for the issue of dropout data, researchers hold different views on its treatment, such as the adjustment of sequencing depth weights [119,120]. Recently, a novel imputation framework called scZN was developed. This framework assumes scRNA-seq data as a combination of the 2-state transcription process and technical dropout, effectively distinguishing true gene silencing from dropout signals, thereby helping present gene expression states more accurately [121]. The integration of scRNA-seq with other existing technologies can also help bridge some of its inherent limitations. For example, performing scRNA-seq after flow cytometric cell sorting can help improve sample preparation qualification rates and effectively enrich low-abundance cell types [122]. In single-cell technology application of benign disease exploration, high sequencing costs restrict longitudinal or multisite sampling, limiting insights into cellular dynamics and spatial organization. Moreover, given the relatively low correlation between mRNA and protein abundance, integrating multi-omics data—including proteomic, microbiomic, epigenomic, and spatial dimensions—is important [123,124]. Yet, the complexity and scale of such multi-omics datasets demand more efficient analytical tools and standardized integration frameworks [23,125]. Progress in hardware (higher-resolution sequencing and imaging) and software (artificial intelligence [AI]-based feature extraction and dimensionality reduction) will be crucial for deciphering single-cell pathological mechanisms.

In the field of BUADs, current treatment remains largely symptomatic and supportive, with limited efficacy. Integrating single-cell and spatial transcriptomic analyses of these diseases enables better identification of key cell subpopulations and critical pathways driving disease progression, guiding subsequent in vitro and in vivo experimental interventions and validation. For example, Peng et al. [35] analyzed bladder tissue from HIC patients using scRNA-seq and found that *TLR3* and *NR2F6* may play important roles in *UPK3A*^+^ umbrella cell injury. Subsequent in vitro and in vivo experiments demonstrated that the TLR3–NR2F6 axis is a potential therapeutic target for HIC [35]. Furthermore, other studies have suggested/identified disease-related targets, such as LBH as a potential biomarker for cavernosal pericytes positively correlated with erectile function [91] and PRMT1 as a biomarker and potential therapeutic target for CaOx-induced kidney injury [50]. However, large gaps still remain in the application of single-cell sequencing in BUADs. Specifically, scRNA-seq remains underutilized in exploring disease areas like urinary system tuberculosis, chronic upper urinary tract infections, urethra stricture, PD, and varicocele. Moreover, domains like ureteral stricture pathophysiology, neuro-immune–muscle interactions in bladder inflammatory or functional disorders, NO–cGMP-independent pathways in ED, and the advancement of systemic diseases like lupus in urological contexts require thorough investigation from the perspective of single-cell sequencing. The application of SRT is even more limited. Future research should emphasize how to obtain ideal disease tissues, how to construct ideal and reliable in vivo and in vitro disease models, and how to apply organoid technology to the BUAD field. Future studies should also attempt to use and enhance relatively economical approaches, such as AI frameworks like SpalM, to leverage scRNA-seq data for predicting and complementing unmeasured genes in spatial transcriptomics data, thereby generating more realistic gene expression maps that reflect in situ spatial distribution [118]. Future research should also incorporate single-cell omics approaches into current research frameworks. For instance, further analysis discovered potential BUAD targets via scRNA-seq [58]. Integrating flow cytometry with scRNA-seq can provide dual-level validation at the RNA and protein stages, thereby boosting biological interpretability, and may facilitate the acquisition and purification of specific rare cell subtypes (like neural cells) for scRNA-seq analysis targeting neural regulation in diseases like IC/BPS, OAB, and ED [122]. For single-cell protein analysis, platforms like BD AbSeq allows concurrent measurement of mRNA and proteins within individual cells [71]. A particular study leveraged the BD AbSeq platform to categorize T-cell subsets in peripheral blood from CP/CPPS patients, uncovering the high proportion of effector T cells, which was confirmed via flow cytometry, thus reinforcing the biological interpretability of the results [71]. Future research should also attempt multi-omics analysis at the single-cell level and build publicly accessible multi-omics databases. A study integrating single-nucleus assay for transposase-accessible chromatin sequencing and chromatin immunoprecipitation sequencing with scRNA-seq and SRT elucidated the epigenetic regulation mechanism of the Nfix–Ifi27 pathway and the antioxidant genes *Sod1*/*Sod2* in PTCs in renal fibrosis, providing a more detailed depiction of the pathological regulatory network [126]. Moreover, techniques such as single-cell genome sequencing and whole-exome sequencing can detect gene mutations and copy number variations, which would be useful for analyzing genetic variation characteristics in congenital diseases and reproductive system disorders [127,128]. For example, a research team conducted whole-exome sequencing and identified a *CCDC149* mutation in a patient with bilateral cryptorchidism, and subsequent scRNA-seq analysis suggested that *CCDC149* may be crucial for normal testicular descent through associations with anti-Müllerian hormone and muscle-related factors [128].

The scope of single-cell sequencing utilization in BUADs ought to be widened. For instance, in infectious diseases, employing pathogen gene detection technologies like INVADE-seq and BacDrop may be beneficial to probe signal cross talk between bacteria and cells in the microenvironment, along with drug resistance mechanisms, helping develop personalized antibiotic or probiotic treatment strategies [95,129]. Future research on BUADs should further obtain temporal dynamic information on disease onset and progression, providing key causal clues. Multi-time-point sequencing can reveal dynamic changes in disease, identify altered subpopulations, and help evaluate key intervention targets. For example, in urolithiasis, multi-time-point single-cell sequencing can be used to study the temporal dynamics between stone formation and changes in the local cellular microenvironment, to better identify key damaged cell subtypes (*HAVCR1*-expressing PTCs) and key intervention targets (CCL2) [12]. It is also crucial to expand the fundamental paradigms and perspectives of current research and actively explore other fields. For example, researchers could investigate the role of key circadian pathways in the immune mechanisms of BUADs, the role of environmental pollutants in BUAD pathogenesis, or the novel mechanisms of old drugs in BUADs [130–132]. Another example is a research team that investigated the “oral–prostate axis” to explore how oral factors regulate apoptosis and proliferation in prostate cells during BPH progression [133,134].

## Conclusion

Parenchymal cell injury and microenvironment remodeling play profound roles in BUAD progression. The application of single-cell and spatial transcriptomics technologies to BUADs overcomes the limitations of traditional histology and molecular biology. These technologies offer novel insights into cellular-level pathological mechanisms and guide further wet-lab validation. Although substantial knowledge gaps remain in the research of BUADs, advances in single-cell techniques, tissue acquisition and processing, multi-omics integration, and computational analysis are expected to enable single-cell technologies to play a pivotal role in therapeutic target discovery and clinical translation, thereby driving the field toward precision medicine.
