# Supplementary material for: Benign Uroandrological Ecosystem: A Thorough Overview from Single-Cell and Spatial Transcriptomics
Source: Research (Wash D C). 2026 Sep 23;9:1438. doi: 10.34133/research.1438 (PMC13598363; doi:10.34133/research.1438)
Supplement: Supplementary file 1 [file research.1438.f1.zip › Supplementary Figure1.docx]

**Supplementary Figure 1.** Features of Bulk RNA-seq, scRNA‑seq and Spatial Transcriptome. RNA sequencing plays a crucial role in the study of benign urological and andrological diseases. In bulk RNA-seq (A), cell mixtures are directly processed for reverse transcription, cDNA amplification and sequencing. In scRNA-seq (B) or spatial transcriptomics (C), we can obtain more information about RNA at the cellular or spatial level within tissues. The figure was created by Figdraw (figdraw.com).
