# Supplementary material for: Benign Uroandrological Ecosystem: A Thorough Overview from Single-Cell and Spatial Transcriptomics
Source: Research (Wash D C). 2026 Sep 23;9:1438. doi: 10.34133/research.1438 (PMC13598363; doi:10.34133/research.1438)
Supplement: Supplementary file 1 [file research.1438.f1.zip › Supplementary Table1.docx]

| Supplementary table 1. The characteristics of core included studies. | | | | | | |
| --- | --- | --- | --- | --- | --- | --- |
| Author | Year | Article type | Journal | PMID/DOI | Study subject/disease | Key sequencing tools |
| Feng et al | 2024 | Review | Military Medical Research | 38605399 | scRNA-seq | - |
| Wang et al | 2025 | Review | Science China Life Sciences | 39792333 | scRNA-seq and SRT | - |
| Anne et al | 2022 | Article | Neurourology and Urodynamics | 36066087 | Social burden of noncancerous genitourinary conditions | - |
| Fink et al | 2022 | Article | Developmental cell | 35914526 | Ureter | scRNA-seq and SRT |
| Ding et al | 2025 | Article | Journal of Inflammation Research | 40979497 | Ureteral stricture | scRNA-seq |
| Guo et al | 2026 | Article | Frontiers in Immunology | 41924262 | Ureteral stricture | scRNA-seq |
| Wang et al | 2022 | Article | Cell Death & Disease | 35941120 | Renal fibrosis | scRNA-seq |
| Paul et al | 2025 | Meeting abstract | Journal of the American Society of Nephrology | 10.1681/ASN.20256xqsvpds | Obstructive nephropathy | SRT |
| Duan et al | 2024 | Article | Advanced Science | 39498889 | Urolithiasis | scRNA-seq |
| Yuan et al | 2025 | Article | Cell Death & Disease | 40744915 | Urolithiasis | scRNA-seq |
| Canela et al | 2023 | Article | Nature Communications | 37468493 | Urolithiasis | snRNA-seq and SRT |
| Jin et al | 2024 | Article | Research | 40330661 | Urolithiasis | scRNA-seq |
| Matković et al | 2026 | Article | Scientific Reports | 41840076 | Urinary bladder | SRT |
| Liu et al | 2025 | Article | PLOS Pathogens | 41042739 | Bladder infection | scRNA-seq |
| Kuret et al | 2025 | Review | BJUI Compass | 40765664 | Interstitial cystitis/bladder pain syndrome | - |
| Peng et al | 2022 | Article | Signal Transduction and Targeted Therapy | 35589692 | Interstitial cystitis/bladder pain syndrome | scRNA-seq and SRT |
| Peng et al | 2024 | Article | The Journal of Pathology | 38551071 | Hunner-type  interstitial cystitis/bladder pain syndrome | scRNA‐seq |
| Zhou et al | 2023 | Article | Frontiers in Immunology | 36814917 | Cystitis glandularis | scRNA‐seq |
| Chen et al | 2024 | Article | MedComm | 38414668 | Bladder outlet obstruction & detrusor underactivity | scRNA‐seq |
| Li et al | 2025 | Article | Computers in Biology and Medicine | 40618693 | Neurogenic bladder | scRNA‐seq |
| Wang et al | 2025 | Article | Advanced Science | 41355531 | Neurogenic bladder | scRNA‐seq |
| Zhang et al | 2025 | Article | Phytomedicine | 39922151 | Overactive bladder | snRNA-seq |
| Yan et al | 2022 | Article | Clinical and Translational Medicine | 36245324 | Prostate | scRNA-seq |
| Fei et al | 2024 | Article | Journal of Translational Medicine | 38654277 | Benign prostatic hyperplasia | scRNA-seq and SRT |
| Shen et al | 2025 | Letter | Genes & Diseases | 40520997 | Benign prostatic hyperplasia | scRNA-seq and SRT |
| Li et al | 2025 | Article | Aging Cell | 40716051 | Benign prostatic hyperplasia | scRNA-seq |
| Zhang et al | 2020 | Article | Journal of Cellular and Molecular Medicine | 33124198 | Chronic prostatitis/chronic pelvic pain syndrome | BD™ AbSeq |
| Zhang et al | 2024 | Article | ImmunoTargets and Therapy | 38435982 | Chronic prostatitis/chronic pelvic pain syndrome | scRNA-seq |
| Jin et al | 2024 | Article | Cell communication and signaling | 39633456 | Chronic prostatitis-like symptoms | scRNA-seq reanalysis |
| Jasmine et al | 2025 | Article | Mucosal immunology | 40907791 | Urethra | scRNA-seq and SRT |
| Henry et al | 2018 | Article | Cell reports | 30566875 | Prostate and prostatic urethra | scRNA-seq |
| Li et al | 2023 | Article | Biochemistry and Biophysics Reports | 36748064 | Urethral stricture | scRNA-seq |
| Zhang et al | 2024 | Article | Journal of Inflammation Research | 39157587 | Urethral stricture | scRNA-seq |
| Oliveira et al | 2025 | Article | Journal of Translational Medicine | 41345677 | Urethral stricture | SRT |
| Milenkovic et al | 2022 | Article | European Urology Focus | 33962884 | Peyronie's disease | scRNA-seq |
| Ilg et al | 2024 | Article | The Journal of Sexual Medicine | 38383071 | Peyronie's disease | - |
| Zhao et al | 2022 | Article | Nature Communications | 35879305 | Corpus cavernosum | scRNA-Seq |
| Yin et al | 2024 | Article | Cell Reports | 39299236 | Corpus cavernosum | scRNA-seq and SRT |
| Chen et al | 2023 | Article | Nature Communications | 37353497 | Erectile dysfunction | snRNA-seq |
| Bae et al | 2024 | Article | eLife | 38856719 | Erectile dysfunction | scRNA-seq and SRT |
| Zhang et al | 2025 | Article | Frontiers in Endocrinology | 41234230 | Erectile dysfunction | scRNA-Seq |
| Li et al | 2022 | Article | Frontiers in Immunology | 35111154 | Orchitis | scRNA-Seq |
| Zhou et al | 2025 | Article | iScience | 41438041 | Testis | INVADE-seq |
| Wang et al | 2025 | Article | The Journal of Clinical Investigation | 41031892 | Epididymo-orchitis | scRNA-seq |
| Cui et al | 2025 | Article | Nature Aging | 40033047 | Aging testis | scRNA-seq |
| Huang et al | 2023 | Article | Protein & Cell | 36929025 | Aging testis | snRNA-seq |
| Zhao et al | 2020 | Article | Nature Communications | 33173058 | Azoospermia | scRNA-seq |
| Huang et al | 2025 | Article | Clinical and Translational Medicine | 41251087 | Aging testis | scRNA-seq and SRT |
| Song et al | 2022 | Article | Nature Communications | 36443325 | Diabetic testis | scRNA-seq |
| Du et al | 2025 | Case report | Andrology | 40459248 | Cryptorchidism | Whole-exome sequencing and scRNA-seq |
| Wang et al | 2024 | Article | Cell Reports Medicine | 39226895 | Cryptorchidism | scRNA-seq |
| Jiang et al | 2025 | Review | Frontiers in Endocrinology | 40177631 | Single-cell sequencing in non-obstructive azoospermia | - |
| scRNA-seq, single-cell RNA sequencing; SRT, spatially resolved transcriptomic | | | | | | |
